# Intrinsic Sensing Properties of Chrysotile Fiber Reinforced Piezoelectric Cement-Based Composites

**DOI:** 10.3390/s18092999

**Published:** 2018-09-07

**Authors:** Jianlin Luo, Chunwei Zhang, Lu Li, Baolin Wang, Qiuyi Li, Kwok L. Chung, Chao Liu

**Affiliations:** 1 School of Civil Engineering, Qingdao University of Technology, Qingdao 266033, China; kuaile8lulu@126.com (L.L.); lqyxyn@163.com (Q.L.); klchung@qut.edu.cn (K.L.C.); alexlc@163.com (C.L.); 2 Collaborative Innovation Center of Engineering Construction and Safety in Shandong Blue Economic Zone, Qingdao University of Technology, Qingdao 266033, China; 3 Center for Infrastructure Engineering, School of Computing, Engineering and Mathematics, Western Sydney University, Sydney, NSW 2751, Australia; b.wang@westernsydney.edu.au; 4 Department of Architecture Engineering, Dezhou Technology Vocational College Qingdao Campus, Qingdao 266232, China; 5 School of Architecture Engineering, Qingdao Agricultural University, Qingdao 266109, China

**Keywords:** structural health monitoring, intrinsic sensor, piezoelectric cement-based composite, chrysotile fiber reinforcing, piezoelectric property

## Abstract

Lead-zirconate-titanate (PZT) nanoscale powder was first synthesized by the sol-gel method, then PZT and 0–3 type PZT/chrysotile fiber (CSF)/cement composite (PZTCC) wafers were fabricated after grind-mixing PZT powder with strontium carbonate and/or cement, ductile CSF in tandem with press-sintered process, respectively. The crystal structure (XRD), microstructure (SEM), piezoelectric properties after surface silver penetration, and polarization of the PZT and PZTCC wafer were investigated. Furthermore, self-sensing responses under either impulse or cyclic loading and micro-hardness toughness of PZTCC were also investigated. Results show that the incorporation of CSF and cement admixture weakens the perovskite crystalline peak of PZTCC; reduces the corresponding piezoelectric coefficient from 119.2 pC/N to 32.5 pC/N; but effectively bridges the gap on the toughness between PZTCC and concrete since the corresponding microhardness with 202.7 MPa of PZTCC is close to that of concrete. A good linear and fast electrical response against either impulse or cyclic loading of the PZTCC is achieved with their respective sensitivity, linearity, and repeatability to 1.505 mV/N, 2.42%, and 2.11%. The sensing responses and toughness of PZTCC is encouraging as an intrinsic piezoelectric sensor for real-time health monitoring of ductile concrete structures.

## 1. Introduction

The monitoring of the structural integrity of concrete structures under external static/dynamic loads ideally employ cement-embedded sensors with fast but highly-accurate responses [1,2,3,4]. As firstly documented by Chung et al. in 1993 [5], cement-based composites incorporating microscale or nanoscale conductive additives, such as steel fibers, carbon fibers [1,5,6,7], carbon blacks [8,9], nickel powders [10,11], semi-conducting nanoparticles [12], carbon nanotubes (CNTs) [1,13,14,15,16,17,18,19], carbon nanofibers [19,20], and graphene [21], have been intensively explored as intrinsic embedded sensors for detecting structural stress/strains via variations in their electrical parameters. However, the polarizations arising from the considerable existing pore electrolytes, in porous cement-based sensors, have significant detrimental effects on the stability and accuracy of the embedded sensors’ electrical responses, even if these sensors are encapsulated [1,6,9]. Han et al. [22] showed that the piezoresistive sensitivity of CNT/cement composites declined with increasing water content. Kim et al. [23] improved sensitivity and stability of piezoresistive sensors for CNT/cement composites after excluding moisture.

Piezoelectric sensors, like BaTiO_3_, KNbO_3_, lead-zirconate-titanate (PZT), and polyvinylidene difluoride-based sensors are insensitive to humidity, and have fast responses and high-accuracies [24,25,26,27,28,29]. Amongst them, the PZT-type sensor has the highest piezoelectric actuating-sensing function and strain sensitivity. Being gain intensive, they have extensive applications in structural health monitoring (SHM) [26,27,28,29]. Song et al. [26] employed two low-cost PZT-based transducers as actuator-receivers of swept sine stress wave, and achieved a good relationship between the damage index, established via wavelet packet analysis, and the loading displacement of L-shaped concrete-filled variable thickness steel tube columns under cyclic loading. However, there are mismatch on the mechanical impedance responses obtained from a pure PZT-type sensor with the concrete when embedded in concrete structures. Furthermore, a pure PZT-type sensor has high density but low ductility, whose interfaces with concrete tend to fail in harsh environments under complicated loadings [27].

Li et al. first introduced a cement admixture into pure PZT to fabricate a piezoelectric cement-based composite (PCM) to compensate for these disadvantages in 2002 [30]. Dong et al. [31,32], Lan et al. [33], Huang et al. [34], Chaipanich et al. [35,36,37], Gong et al. [38,39], Pan et al. [40,41], Luo et al. [42], and Zhang et al. [43] reported that, compared with 1–3, 2–2 type PCM sensor [31,33], the 0–3 type PCM sensor had more stable piezoelectric self-sensing behaviors with age and temperature treatment, superior compatibility and impedance-matching with concrete, where PZT granules were directly but randomly distributed in three dimension cement matrix without extra cutting and embedding processes [31,41]. Moreover, PZT powder in nanoscale can be facile pressed and sintered into PCM-based sensor or actuator for SHM with accurate stoichiometric composition, uniform morphology, and proper microstructure to ensure good interface compatibility and enhanced piezoelectric sensitivity [38]. However, the ductility of PCM is still relatively low especially where PCM sensors are embedded into long slim concrete structures comprised of fiber reinforced concretes, or ductile engineering cementitious composites [44].

The fabrication processes of the PZT nanoscale powder is vital to the performance of 0–3 type PCM sensor. Compared with the synthesis processes of mechanochemical [45,46], microwave radiation [47], hydrothermal [48], and coprecipitation [49], the sol-gels process is the most widely-used synthesis process for PZT nanoscale powder due to its resulting uniform composition, accurate stoichiometric ratio, and ultra-high purity under laboratory operation [50,51,52]. Mu et al. developed a modified sol-gel method with water and a little absolute ethanol as solvents and inorganic salts as raw materials to achieve a uniform single-phase PZT powders with an average size of 70 nm after annealing for 2.5 h at 700 °C [51].

In this study, PZT nanoscale powder was first synthesized by the sol-gel method [51]. A trace of strontium carbonate was introduced to diminish shrinkage and stabilize crystal phase during PZT wafer calcination process [53]. Furthermore, ductile chrysotile fiber (CSF) was added into the PZT/cement grind-mixture to fabricate the 0–3 type PZT/CSF/cement composite (PZTCC) wafer by press-sintering method [54]. Thirdly, the piezoelectric, toughness, and self-sensing properties of PZT and PZTCC wafers were characterized to develop a type of intrinsic and ductile piezoelectric sensor for SHM in complex structure with balanced piezoelectric sensitivity and toughness.

## 2. Materials and Methods

### 2.1. Raw Materials

Lead acetate (PbC_4_H_6_O_4_·3H_2_O, mass fraction ≥ 99.5%), zirconyl nitrate (ZrO(NO_3_)_2_, mass fraction ≥ 99.5%), tetrabutyl titanate (Ti(OC_4_H_9_)_4_, mass fraction ≥ 98%) were all purchased from Shanghai Tongya Chemical Technology Development Co., Ltd. (Shanghai, China); Strontium carbonate (SrCO_3_, mass fraction ≥ 99.5%) was purchased from Sigma-Aldrich (Shanghai, China) Trade Co. Ltd. (Shanghai, China); Ethylene glycol (HOCH_2_CH_2_OH, mass fraction ≥ 96%), glacial acetic acid (CH_3_COOH, mass fraction ≥ 99.5%), ammonia (NH_3_·H_2_O, mass fraction = 25–28%) were all obtained from Yantai Sanhe Reagent Co. Ltd. (Yantai, China); Dimethicone ((C_2_H_6_OSi)_n_, AR) and alumina (Al_2_O_3_, AR) were both obtained from Tianjin Yongda Reagent Co. Ltd. (Tianjin, China); Polyvinyl alcohol was obtained from Sinopharm Group Chemical Reagent Co., Ltd. (Shanghai, China); Conductive silver paste (DAD-87 type) was purchased from Shanghai Synthetic Resin Research Institute (Shanghai, China); Anhydrous ethanol (C_2_H_5_OH, mass fraction ≥ 99.7%) was obtained from Shanghai Ebi Chemical Reagent Co., Ltd. (Shanghai, China); Distilled water was commercially available; Cement was purchased from Shanshui Cement Group Co., Ltd. (P.O. 52.5 type, Qingdao, China); CSF was obtained from Qinghai Chuangan Co. Ltd. (Mangya, China).

### 2.2. Preparation of PZT Nanoscale Powders, PZT Wafer, and PZTCC Composite

**Figure 1:**
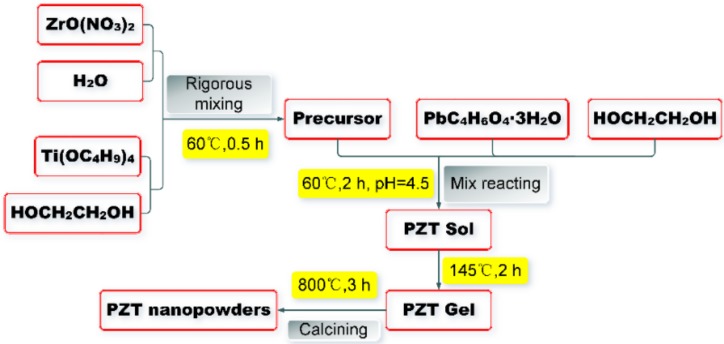
The sol-gel synthesis schematic procedure for lead-zirconate-titanate (PZT) nanoscale powders.

The sol-gel schematic method for synthesizing PZT nanoscale powders was demonstrated in Figure 1. Lead acetate, zirconyl nitrate, and tetrabutyl titanate were weighed to molar ratio for *r*(Pb)/*r*(Zr)/*r*(Ti) to 1:0.52:0.48. The resulting zirconyl nitrate–tetrabutyl titanate mixture was dissolved in 50 mL of water with a small amount ethylene glycol, and rigorously mixed together for 0.5 h at 60 °C in a collector thermostatic magnetic stirrer (DF-101S type, Zhengzhou Keda Machinery And Instrument Equipment Co., Ltd., Zhengzhou, China) filled with dimethicone to prepare the precursor. Then, the lead acetate was dissolved in ethylene glycol where *c*(Pb^2+^) was 15% [51], and slowly added to the abovementioned precursor. Glacial acetic acid and ammonia was used to adjust the pH value to around 4.5, and then reacted for another 2 h at 60 °C to fabricate the PZT sol. The PZT sol was dried at 100 °C, and residual organics were incinerated in a resistance furnace to form the pre-sintering PZT. The pre-sintered PZT was sintered for 2 h at 800 °C in a high-temperature furnace (XZK-3 type, Longkou Electric Furnace Factory, Longkou, China) [34]. The PZT nanoscale powder was obtained by grinding.

The PZT nanoscale powder, strontium carbonate (0.5% of PZT amount), for crystalline stabilization and deformation suppression, were glued together with polyvinyl alcohol and compressed into a wafer using a powder metallurgy tablet machine (HY-2H type, Tianjin Sinteron Powder Metallurgy Co., Ltd., Tianjin, China) for 2 min at 12 MPa. The PZT wafer had its glue removed by heating for 2 h at 300 °C, and sintered for another 2 h at 800 °C with a surface covering alumina powder. The sintered PZT wafer was cleaned with anhydrous ethanol before a silver paste overlay was twice applied then heated for 3 h at 100 °C. A 3000 kV/cm polarized voltage (DW-6000DC type, Tianjin Dongwen High Voltage Company, Tianjin, China) was applied for 30 min to the PZT wafer soaking in 120 °C dimethicone solution [37].

**Figure 2:**
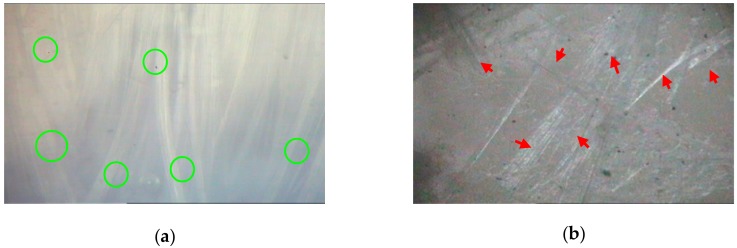
The optical morphologies of chrysotile fiber: (**a**) in bundle (green circles) (×200); (**b**) dispersed state in ethanol (red arrows) after grinding for 30 min (×200).

The PZT nanoscale powder and strontium carbonate was ground together. CSF (0.05%) and cement (25% of PZT amount) was then added to the grinding process to create the PZT/CSF/cement (PZTCC) mixture, using anhydrous ethanol as a dispersing aid. An optical microscope (OP, XC4 type, Shanghai Precision Optoelectronics Co. Ltd., Shanghai, China) was used to observe the CSF in bundle and dispersed in anhydrous ethanol (Figure 2). The PZTCC mixture was compressed, glue removed, sintered, silver penetrated, and polarized as described in the PZT wafer preparation. It is worth noting that the PZTCC wafer, after sintering, was hydrated with a close fitting wet cotton wick, and oven-dried before silver penetration.

### 2.3. Characterization of PZT Nanoscale Powders, PZT Wafer, and PZTCC Composite

X-ray powder diffraction (XRD, D8 Advance type, Bruker AXS Inc., Karlsruhe, Germany) and scanning electronic microscopy (SEM, S3500N type, Hitachi Co., Ltd., Tokyo, Japan) were used to characterize crystalline structures, particle sizes, and microstructures of PZT powder and PZTCC composites.

After surface polishing with different mesh sand papers, microhardness toughness measurement (HVS-1000 type, Laizhou Lyric Testing Equipment Co., Ltd., Laizhou, China) of the PZTCC composite was taken under a 1000 g load at 20 s compress duration. A quasi-static instrument (ZJ-6A type, Institute of Acoustics, CAS, Beijing, China) measured the *d*_33_ coefficients of PZT and PZTCC wafer after polarization.

A dynamic data acquisition system (DASP-V10 type, Orient Institute of Noise & Vibration, Beijing, China) measured the electricity responses versus hammer impulses at various loading levels (100 N, 200 N, 300 N, 400 N) of PZTCC wafer with the top and bottom surfaces connected to a lead wire by conductive silver paste. DASP-V10 data acquisition system in tandem with a universal testing machine (E43 type, SANS-MTS, MTS Systems (China) Co., Ltd., Shenzhen, China) collected data from the applied loads and voltage responses of PZTCC wafer against time, and investigated the self-sensing property of PZTCC under cyclic loading with 0–100 N range at 2 N/s head speed.

## 3. Results and Disscussion

### 3.1. The Crystalline Structures of PZT and PZTCC Composite

**Figure 3:**
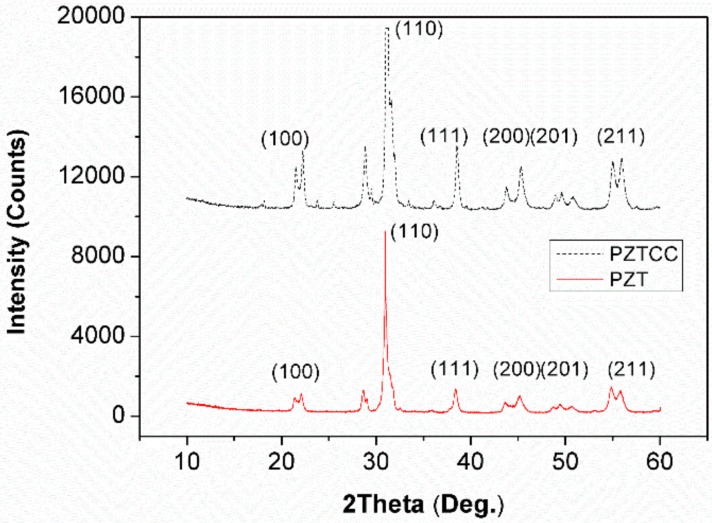
XRD crystallinity of PZT and PZTCC (PZT/CSF/cement composite).

Figure 3 demonstrates the crystalline structures of PZT and PZTCC composite.

Figure 3 shows characteristic peaks for PZT (2*θ* = 21.3°, 31.0°, 38.4°, 45.2°, 49.5°, and 54.8°), which represent the (100), (110), (111), (200), (201), and (211) patterns of PZT sintering for 2 h at 800 °C. Trace strontium carbonate shows no effect on the crystalline structure of PZT and the main peak (110) of PZT is Pb(Zr_0.52_Ti_0.48_)O_3_ crystal phase, which is the classic ABO_3_ type perovskite [53]. Although the addition of cement and CSF in PZTCC is insignificant on the basic crystal structures of PZT, several miscellaneous peaks appear, and their corresponding main peak (110) is also reduced, contributing to an inferior perovskite structure due to cement and CSF introduction (Figure 3).

### 3.2. Microstructure and Piezoelectric Coefficient of PZT and PZTCC Composite

**Figure 4:**
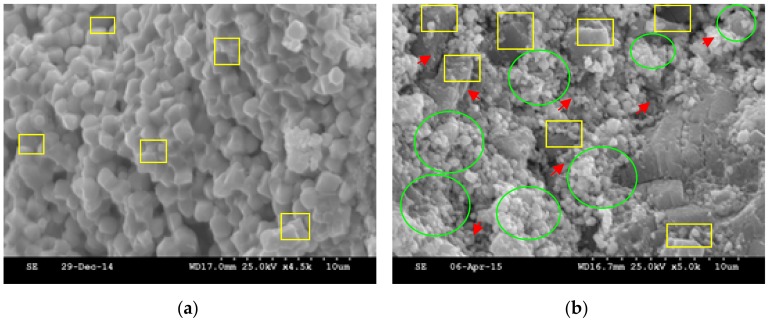
SEM images of PZT and PZTCC composite: (**a**) PZT (yellow rectangle, PZT); (**b**) PZTCC (yellow rectangle, PZT; green ellipse, cement particle; red arrow, CSF).

Fine and even cubic crystallizations of PZT are observed in Figure 4a, the crystal sizes are narrowly distributed in the 900–1100 nm range [38]; the corresponding *d*_33_ coefficient is up to 119.2 pC/N. In Figure 4b, CSF and cement without any piezoelectric effect are dispersed within the PZT perovskite crystals, and perovskite PZT crystals are comparatively larger than those in the PZT wafer, which reduces the corresponding piezoelectric macro-performance of the PZTCC wafer, whose d_33_ coefficient is only 32.5 pC/N.

### 3.3. Microhardness Toughness Properties of PZTCC Composite

**Figure 5:**
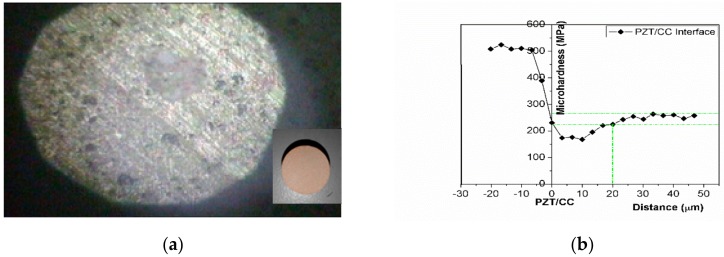
(**a**) An optical microscope image of the surface-polished PZTCC composite (×400) and (the insert bottom-right picture of the PZTCC composite at macro-scale); (**b**) Micro-hardness distributions at the PZT/CC interface.

Figure 5 show surface images of the surface-polished PZTCC composite at macro-scale and as observed in OP. The interface microhardness of the PZTCC composite is 202.7 ± 4.9 MPa and the interface width is 20 ± 0.3 μm. Compact interface connections between PZT nanoscale crystals among the cement matrix are achieved, also verified in Figure 4b. The resultant interface hardnesses are similar to common concrete [55], and suited to applications as embedded intrinsic sensors in concrete structures.

### 3.4. Piezoelectric Sensing Properties of PZTCC Wafer

**Figure 6:**
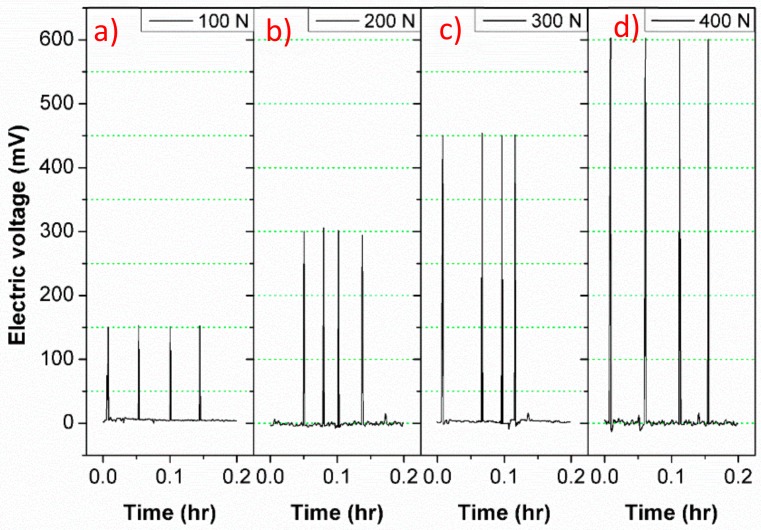
Electric voltage versus time of the PZTCC wafer under various impulse levels: (**a**) 100 N; (**b**) 200 N; (**c**) 300 N; (**d**) 400 N.

**Figure 7:**
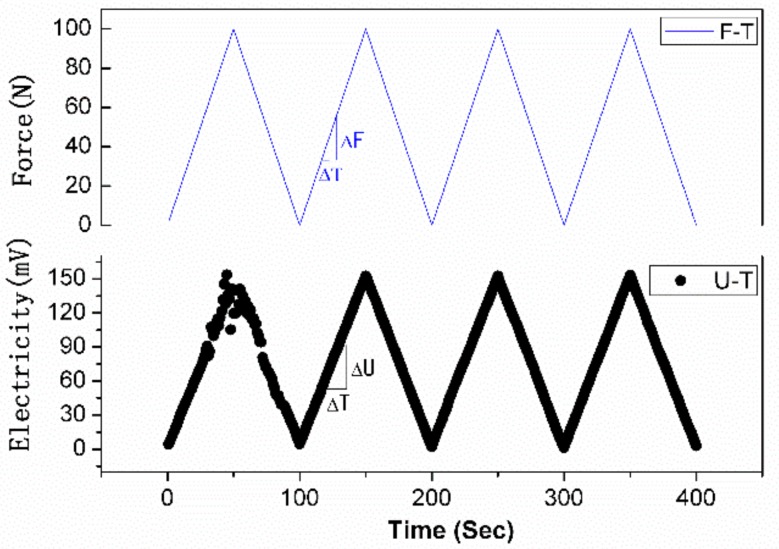
Electricity history of PZTCC wafer under cyclic loading.

The electrical voltage time-history of the PZTCC wafer under various impulse levels and cyclic loads are recorded and illustrated in Figure 6 and Figure 7, respectively.

In Figure 6, the electricity output almost retains 150 mV, 300 mV, 450 mV, and 600 mV against their corresponding 100 N, 200 N, 300 N, 400 N impulses, over four repeat tests. It shows both a good linearity and a rapidity of the electrical response to the impluse level, although the *d*_33_ coefficient is relatively lower than that of common PZT wafer [35]. Figure 7 shows that the electrical voltages vary linearly with the applied cyclic force, although there is an initial fluctuations at the initial loading cycle.

The linearity (*e*), sensitivity (*k*) and repeatability (*e*_f_) of the sensor paramters of the PZTCC wafer under cyclic loading can be calculated by the following formulas [1,8],

*e* = ±Δ_max_/U_F.S_ × 100% (1)

*k* = ΔU/ΔF (2)

*e*_f_ = ±Δ′_max_/2U_F.S_ × 100% (3)

The Δ_max_, U_F.S_, ΔU, ΔF, and Δ′_max_ represents the maximum deviation between the electricity (U)- force (F) curve and the linear fitting line, full-scale output of U, change in U, change in F, and maximum deviation in forward and reverse stroke of U-F curve, respectively.

Table 1 shows the calculated *e*, *k*, and *e*_f_ parameters with their corresponding means, derived after excluding the fluctuations of cycle 1.

It can be determined from Figure 7 and Table 1 that, the sensitivity *k* is 1.505 V/kN, which suits most civil engineering applications [1]. Further, both *e* and *e*_f_ parameters are well below 5%, and their correponding means are only 2.42%, 2.11%, respectively. These sensing characteristics make PZTCC wafer suited to applications as a stress/strain sensor with stable linear responses.

Trace additions of strontium carbonate is effective in stabilizing the crystalline structure and retards volume shrinkage during the sintering of the PZT or PZTCC wafer. CSF and cement, as inorganic but fireproof admixture, lower the piezoelectric coefficient of PZTCC, whilst both equalizing the mechanical impedance factor of the PZT with concrete, and improving the fracture toughness of the corresponding PZTCC composite, where the ductile CSF achieved a fully netlike distribution. The overall results on piezoelectric sensitivity and toughness indicate a PZTCC wafer to be well suited as an intrinsic compatible sensor, which can be embedded in concrete structures for in situ monitoring.

## 4. Conclusions

(1) PZT nanoscale powder was successfully synthesized using the sol-gel process, from which a PZT wafer was created by sintering with trace of strontium carbonate producing fine and even cubic crystals within the 900–1100 nm range. CSF and cement were ground together with PZT nanoscale powder using ethanol as a dispersant to fabricate a PZTCC wafer in the same process as with the PZT wafer.

(2) CSF and cement incorporation weakens (110) perovskite crystallinity of PZTCC with cubic crystals, the *d*_33_ reduces from 119.2 pC/N to 32.5 pC/N, but narrows the hardness gap between the PZTCC and concrete, the corresponding microhardness is only 202.7 MPa, and improves intrinsic toughness and impedance-matching with common concrete.

(3) A good linearity and fast response between the electrical signal against the loading of PZTCC wafer in either impulse or cyclic states is achieved with their corresponding sensitivity, linearity, and repeatability to 1.505 mV/N, 2.42%, and 2.11%, respectively.

The *d*_33_ of this type of ductile piezoelectric PZTCC sensor is not high enough to enable in situ monitoring application. In the future, the corresponding piezoelectric property will be greatly improved by aging and temperature treatment method before developing as intrinsic sensor embedded in complex structures for real-time monitoring.

## Figures and Tables

**Table 1:** The linearity (*e*), sensitivity (*k*), and repeatability (*e*_f_) of the PZTCC under cyclic loading.

Parameter	Cycle 1	Cycle 2	Cycle 3	Cycle 4	Mean
*e*	25.94%	3.04%	2.86%	2.41%	2.42%
	16.08%	2.18%	2.01%	2.02%	
*k*	1.1751	1.5080	1.4973	1.5132	1.505 mV/N
	1.3844	1.5064	1.5049	1.5003	
*e*_f_	2.4%	2.4%	1.5%	2.42%	2.11%

## Nomenclature

PZT	Lead-zirconate-titanate
XRD	X-ray powder diffraction
SEM	Scanning electronic microscopy
OP	Optical microscope
CSF	Chrysotile Fiber
SHM	Structural health monitoring
PZTCC	PZT/CSF/cement composite
*d*_33_	Piezoelectric coefficient
*e*_f_	Repeatability
U_F.S_	Full-scale output of electricity (U)
Δ_max_	Maximum deviation between U- force (F) curve and the linear fitting line
Δ′_max_	Maximum deviation in forward and reverse stroke of U-F curve
